# *In vitro* schistosomicidal activity of the lignan (−)-6,6′-dinitrohinokinin (DNHK) loaded into poly(lactic-co-glycolic acid) nanoparticles against *Schistosoma mansoni*

**DOI:** 10.1080/13880209.2017.1405996

**Published:** 2017-11-26

**Authors:** Thaís C. Lima, Rodrigo Lucarini, Priscilla P. Luz, Emerson H. de Faria, Liziane Marçal, Lizandra G. Magalhães, Fernanda R. Badoco, Viviane R. Esperandim, Eduardo F. Molina, Rosangela S. Laurentz, Regiane G. Lima, Wilson R. Cunha, Jairo K. Bastos, Marcio L. Andrade Silva

**Affiliations:** a Laboratório de Pesquisa em Microbiologia Aplicada, Núcleo de Ciências Exatas e Tecnológicas, Universidade de Franca, Franca, Brazil;; b Departamento de Química–CCE, Universidade Federal do Espírito Santo (UFES), Vitoria, Brazil;; c Faculdade de Engenharia de Ilha Solteira, Universidade Estadual Paulista, Ilha Solteira, Brazil;; d School of Pharmaceutical Sciences of Ribeirão Preto, University of São Paulo, Ribeirão Preto, Brazil

**Keywords:** PLGA, biological activity, nanoparticulate formulation, 6,6′-dinitrohinokinin, schistosomicidal

## Abstract

**Context:** (−)-6,6′-Dinitrohinokinin (DNHK) display remarkable antiparasitic activity and was, therefore, incorporated into a nanoparticle formulation.

**Objective:** Incorporation of DNHK in poly lactic-co-glycolic acid (PLGA) nanoparticles aiming to improve its biological activities.

**Materials and methods:** Synthesis, characterization and incorporation of DNHK into glycolic acid (PLGA) nanoparticles by nanoprecipitation method. The nanoparticles were characterized by ultraviolet-visible spectroscopy, X-ray diffraction, field emission electron microscopic scanning *mansoni* (FESEM), and dynamic light scattering (DLS). For the *in vitro* test with *Schistosoma mansoni*, the DNHK-loaded PLGA was diluted into the medium, and added at concentrations 10–200 µM to the culture medium containing one adult worm pair. The parasites were kept for 120 h and monitored every 24 h to evaluate their general condition, including: pairing, alterations in motor activity and mortality.

**Results:** The loaded PLGA nanoparticles gave an encapsulation efficiency of 42.2% and showed spherical characteristics in monodisperse polymeric matrix. The adult worm pairs were separated after 120 h of incubation for concentrations higher than 50 µM of DNHK-loaded PLGA. The groups incubated with 150 and 200 µM of DNHK-loaded PLGA for 24 and 120 h killed 100% of adult worms, afforded LC_50_ values of 137.0 ± 2.12 µM and 79.01 ± 1.90 µM, respectively, which was similar to the effect displayed by 10 µM of praziquantel.

**Discussion and conclusions:** The incorporation of DNHK-loaded showed schistosomicidal activity and allowed its sustained release. The loaded PLGA system can be administered intravenously, as well as it may be internalized by endocytosis by the target organisms.

## Introduction

Recently, considerable attention has been devoted to the use of nanostructured biodegradable materials for several biomedical applications, such as drug delivery systems, biosensors, biomarkers and molecular imaging (Zhang et al. 2012). In particular, studies in the pharmaceutical field, considering drug delivery systems (DDS), have focused on the improvement of drug release control, such as the specificity and selectivity of releasing, protection of labile active principles from degradation and/or inactivation by the gastric juice, improvement of drug bioavailability through increased cell permeation of hydrophilic substances, as well as the reduction of the necessary drug dosage and the side effects that normally arise during conventional therapy (Puisieux and Roblot-Treupel 1989; Faraji and Wipf 2009; Durán et al. 2010). The sustained drug release at a specific site, time and at defined therapeutic dosages has been the main objective during the design of Drug Delivery System (DDS), such as liposomes, micelles, polymeric micro/nanoparticles and magnetic nanoparticles (Pankhurst et al. 2003; Chen et al. 2010).

The biodegradable polymers are usually chosen as DDS, since they do not have to be removed after implantation. This advantage is due to the fact that they degraded both *in vitro* and *in vivo* either into non-toxic substances that are normal metabolites of the body or into products that can be completely eliminated from the body with or without further metabolic transformations (Nair and Laurencin 2006; Nassar et al. 2011). Gelatin (hydrolyzed form of collagen), chitosan (deacetylated form of chitin) and other polysaccharides are the commonly natural biodegradable polymers used for DDS (Saravanakumar et al. 2012). The most used synthetic polymers are polycaprolactone (PCL), polyethylene glycol (PEG) and the aliphatic polyesters, such as: polylatic acid (PLA), polyglycolic acid (PGA) and their copolymer polylactic-co-glycolic acid (PLGA).

The nanoparticles made by biodegradable polymers have been well studied, which have been found to be interesting vehicles for prolonged drug release at the target site. In addition, the diameter size (between 10 and 1000 nm) elevates blood circulation and the higher physical–chemical stability, in comparison with liposomes, which is also an advantage exhibited by the polymeric nanoparticles (Madaswamy et al. 2009). The polymeric particles have been successfully employed for encapsulation of a wide range of pharmaceuticals and bioactive compounds, such as proteins, enzymes, hormones and vaccines. The incorporation process does not affect their stability and biological activity, allowing achieving high drug incorporation efficiency (Aftabrouchad and Doelker 1992; Rajeev et al. 2000).

The application of DDS for carrying antiparasitic drugs is inexpressive comparing to, for example, chemotherapeutic and anti-inflammatory drugs. Considering only the antiparasitic drugs, the employment of DDS for the treatment of schistosomiasis and trypanosomiasis diseases is fewer than for other parasitic diseases, such as leishmaniasis and malaria. For example, Luz et al. (2012) incorporated curcumin into PLGA nanospheres and evaluated the *in vitro* schistosomicidal activity of curcumin-loaded PLGA nanoparticles against adult *Schistosoma mansoni* Sambon (Schistosomatidae), causing the death of 100% of parasites at 50 and 100 μM after 120 and 24 h, respectively. In addition, separation of the adult worm pair couples, decreasing of the motor activity and partial alterations in tegument of the adult worms were observed.

Other biologically active compounds have been extensively investigated and obtained by our research group, such as (−)-cubebin and its derivatives. These compounds belong to lignan class, which display a wide range of chemical structures and biological activities (Ward 1999). It is noteworthy that over the last two decades natural products have largely contributed to the development of new medicines, since most commercially available antimicrobial and anticancer agents have been directly or indirectly produced from natural active compounds. In general, approximately 40% of the new pharmaceuticals launched into the market are from natural origin or have been obtained based on the natural prototypes (Newman et al. 2003).

(−)-Cubebin, a dibenzylbutyrolactolic lignan, displays weak trypanocidal and moderate anti-inflammatory activities (Bastos et al. 1999; Jeng and Hou 2005). However, derivatives of (−)-cubebin, including (−)-hinokinin, display better trypanocidal activity against the amastigote forms of *Trypanosoma cruzi* (Barreiro and Fraga 2001; Da Silva et al. 2005; Jeng and Hou 2005; Silva et al. 2007, 2009). In addition, the (−)-6,6′-dinitrohinokinin (DNHK) obtained by Silva et al. (2009) by partial synthesis of (−)-hinokinin, displayed schistosomicidal activity against *S. mansoni* in both *in vitro* and *in vivo* (Pereira et al. 2015). Therefore, based on the schistosomicidal effect of DNHK, this work incorporates DNHK in PLGA nanoparticles in an attempt to improve the schistosomicidal activity of this compound.

## Materials and methods

### Materials

Chloroform (CHCl_3_), deuterated chloroform (CDCl_3_), pyridinium chlorochromate (PCC), dichloromethane (DCM), fuming nitric acid (HNO_3_), sodium bicarbonate (NaHCO_3_), methanol (MeOH) and silica gel 60 (0.063–0.200 nm pore size) were purchased from Merck & Co (Kenilworth, NJ). MeOH chromatography (HPLC) grade was provided by J. T. Baker (Philippsburg NJ). Aluminum sheets (20 × 20 cm and 1 mm thick) were acquired from Merck & Co. TLC Silica gel 60 F_254_, PLGA (Mw =50,000–75,000 g mol^−1^), with a lactic acid/glycolic acid ratio of 85:15 and Pluroni F68 were obtained from Sigma-Aldrich. Acetonitrile HPLC grade was purchase from Omnisolv Inc., and the Milli-Q water used in the experiments was obtained from Millipore, Direct-Q 3 UV with pump. ^1^H NMR and ^13^C NMR were obtained to confirm the DNHK chemical structure. Proton nuclear magnetic resonance (^1^H NMR) spectra were acquired on a Bruker AVANCE 300 spectrometer operating at 300 MHz in CDCl_3_ for ^1^H NMR and 75 MHz in CDCl_3_ for ^13 ^C NMR (Supplementary Table S2).

### *Synthesis of* (−)-6,6′-*dinitrohinokinin[(3 R,4 R)-3,4-bis(6-nitro-1,3-benzodioxol-5-yl)methyl)-dihydro-2(3H)-furanone] (DNHK)*

Prior to the DNHK synthesis, (−)-cubebin was isolated from *Piper cubeba* L. f. (Piperaceae) seeds and (−)-hinokinin was obtained by oxidation of (−)-cubebin (Souza et al. 2005). (−)-Hinokinin was then submitted to a nitration reaction in the presence of fuming HNO_3_. The reaction was undertaken under magnetic stirring at 0 °C for 2 h. The obtained DNHK was crystallized as yellowish powder from MeOH. The purity of the synthesized DNHK was verified by HPLC analysis (Setchell et al. 1981; Costa 2000).

### Incorporation of DNHK into PLGA 85:15 nanoparticles by the nanoprecipitation method

The incorporation of the obtained DNHK into PLGA particles was accomplished by nanoprecipitation method described by Fessi et al. (1989), which involves the mixture of an organic phase with an aqueous phase. For that, an organic phase composed of PLGA 85:15 (∼49 mg), acetonitrile (0.23 mol), DNHK (10% w/w) and an aqueous phase composed by Pluronic F68 (10% w/w) and distilled water (0.68 mol) were initially prepared in different flasks. After that, the organic phase was slowly dropped into the stirred aqueous phase at room temperature using a syringe. The resulting suspension was maintained under stirring for 30 min and the acetonitrile was then removed at 50 °C under reduced pressure. The final aqueous suspension was used for further characterizations.

### DNHK purity determination

HPLC analysis was carried out to evaluate the purity of the synthesized DNHK by using aCBM-20 A Schimadzu chromatographer A with a C-18 ELC-ODS column. The mobile phase consisted of 1:1 MeOH/water:pure MeOH in a gradient mode, using a diode array detector (Supplementary Figure S1).

### DNHK-loaded PLGA 85:15 nanoparticles

The encapsulation efficiency of DNHK-loaded PLGA particles was determined by ultraviolet–visible spectroscopy (UV–Vis spectroscopy) using a Hewlett-Packard 8453 Diode Array spectrophotometer. Three aliquots of the aqueous suspension containing DNHK-loaded PLGA particles were placed into centrifuge tubes and submitted to SpeedVac™ concentrator (Savant SPD 2010, Thermo Electron Corporation) in order to remove the water. Then, acetonitrile was added to the samples and the released DNHK was analyzed by UV–Vis spectroscopy. Its concentration was determined based on a standard curve of DNHK in acetonitrile, which was drawn by the standard addition method using known concentrations of DNHK standard solutions in acetonitrile from 2.4 × 10^−5^ to 14.0 × 10^−5 ^mol/L, and their respective absorbance values at 348 nm.

The average particle diameter was analyzed by dynamic light scattering (DLS) on Zetasizer Malvern 3000 HSa equipment. For this purpose, the aqueous suspension obtained in 2.3 was diluted prior to the analysis.

The particles shape was evaluated by Field Emission Scanning Electron (FESEM) Microscope using a Phillips^®^ XL30A microscope. To this end, the aqueous suspension was added dropwise over a glass slide previously fixed on the analysis support. After drying, the samples were covered with a thin gold layer in order to reduce charging and produce a conductive surface.

The samples crystallinity was characterized by X-ray diffraction (XRD) using a Rigaku MiniFlex II Desktop X-ray Diffractometer. The analysis was carried out at 30 kV, 15 mA, using filtered Cu Kα radiation and varying the angle 2θ from 5 to 75°. All the analyzes were processed at a scan speed of 0.04°/s.

### *In vitro* schistosomicidal activity of PLGA-DNHK

Luis Evangelista (LE) strain of *S. mansoni* was maintained by passage through *Biomphalaria glabrata* snails and Balb/c mice. After eight weeks, *S. mansoni* adult worms (male and female) were recovered under aseptic conditions from mice previously infected with 200 cercariae by perfusion of the livers and mesenteric veins (Smithers and Terry 1965). The worms were washed in RPMI 1640 medium (GIBCO), kept at pH 7.5 with HEPES 20 mM and supplemented with penicillin (100 UI/mL), streptomycin (100 µg/mL) and 10% FBS (GIBCO).

For the *in vitro* test with *S. mansoni*, the DNHK-loaded PLGA was diluted into the medium to give 10–200 µM, which were added to the medium containing one adult worm pair after a period of 24 h of adaptation to the culture medium. The parasites were kept for 120 h and monitored every 24 h to evaluate their general condition, including: pairing and alterations in motor activity (Xiao et al. 2007). Worm motility was classified as normal motility, no motility (no movement after 2 min. of observation-dead), decreased motility (decreased motility compared to the negative control groups), and minimal movement (occasional movement of the head and body) either slight or significant (Magalhães et al. 2012). Parasites in RPMI 1640 medium and parasites in RPMI plus PLGA were used as the negative control group, and parasites in 10 µM of praziquantel (PQZ) were used as the positive control group. All experiments were carried out in quadruplicate and repeated at least three times.

### Statistical analysis

All the results are expressed as the mean ± S.E.M. Data were statistically analyzed by one-way analysis of variance followed by Dunnet’s comparison. The statistical tests were performed with the aid of the Graphpad Prism (version 5.0) software (Graphpad Software Inc., La Jolla, CA). Lethal concentration (LC_50_) value was calculated from a non-linear regression dose–response inhibition graph using the same software described above.

## Results and discussion

### DNHK synthesis

**Scheme 1.**
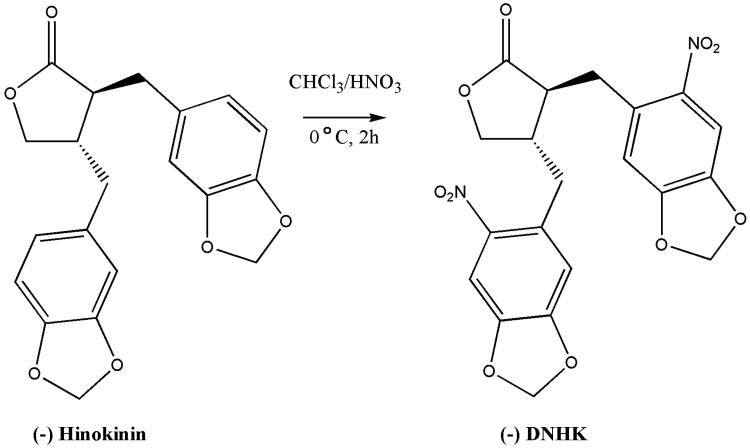
Representation of the DNHK synthesis by nitration of (−)-hinokinin.

The DNHK was obtained from the functionalization of the (−)-hinokinin aromatic rings with NO_2_ groups with 97.6% yield. The synthesis success was confirmed by ^1^H NMR and ^13^C NMR, and the DNHK purity was estimated by HPLC analysis. After 18.05 min, the DNHK started to be detected at 254 nm, and its purity of 95% was determined by the LC Solution software. All spectroscopic data are in accordance with the assumed chemical structures (Supplementary Figures S2 and S3), and literature data (Souza et al. 2005) (Scheme 1). Position of the nitro groups on the aromatic rings was confirmed by the absence of coupling between aromatic protons, which indicates that these groups are bound at positions 6 and 6′ of the aromatic rings (Da Silva et al. 2005).

### DNHK concentration in PLGA 85:15 particles

**Figure 1.**
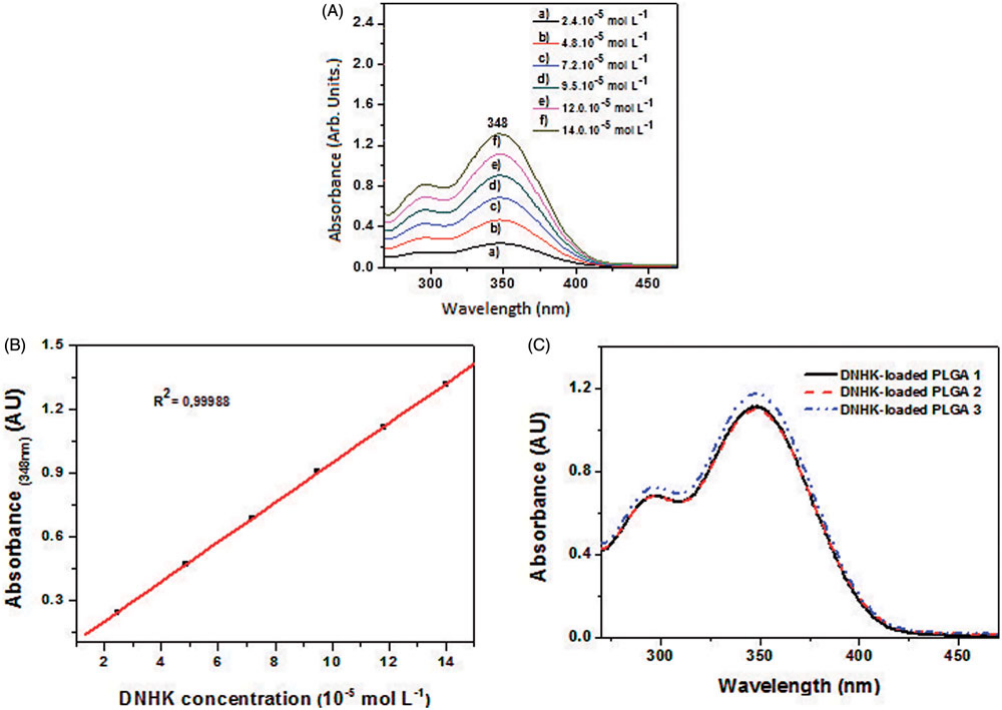
(A) Absorption measurements of DNHK standard solutions by UV–Vis spectroscopy. (B) DNHK standard curve in acetonitrile acquired using the DNHK absorbance values at 348 nm. (C) Electronic absorption spectra of DNHK extracted from the PLGA nanoparticles. The three curves for each fibre indicate the triplicate, and all graphics were obtained by using Origin 8.0 software.

The six standard points curve for DNHK was obtained by the standard addition method, and it was constructed after each DNHK standard solution was analyzed by UV–Vis spectroscopy (Figure 1).

The encapsulation efficiency of DHKN-loaded PLGA nanoparticles was calculated based on the absorption values obtained (Figure 1(C)) and the standard curve (Figure 1(B)). The determined encapsulation efficiency of DNHK was 42.2 ± 1.6%. Saraiva et al. (2010) made the encapsulation of hinokinin in PLGA particles by the emulsion/solvent evaporation method, in which the average diameter of the obtained particles was 0.862 µm, and the attained encapsulation efficiency was approximately 72%. The particles obtained in this work were four times smaller than the particles obtained by Saraiva et al. (2010). Smaller particles tend to present lower encapsulation efficiencies due to their reduced size. Despite of the tinnier particles, the obtained encapsulation efficiency was 42.2%.

**Figure 2.**
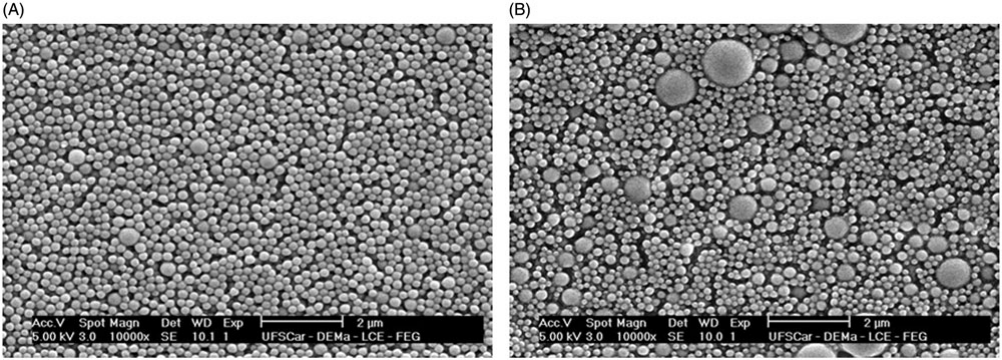
FESEM images of (A) DNHK-loaded PLGA 85:15 and (B) PLGA 85:15 nanoparticles.

The DNHK-loaded PLGA 85:15 nanoparticles morphology was characterized by Field Emission Scanning Electron Microscopy (FESEM) (Figure 2). According to the FESEM micrograph, the produced particles are spherically shaped and appear to have a homogeneous size distribution. After the addition of DNHK, there was a change in the morphology of the particles, making them smaller and more homogeneous.

The DLS confirmed the production of the particles in the nanometric range, since the obtained average diameter was 215 nm, and the homogeneity in the diameter sizes, since the polydispersion index (PI) was 0.102. This low PI (close to zero) indicated the formation of monodispersed particles.

On one hand, concentrations higher than 10% of DNHK-loaded PLGA leads to crystallization of DNHK on the nanoparticles surface, and on the other hand, concentrations lower than 4% leads to the formation of interconnected and inhomogeneous nanoparticles (Figure 2(A)). However, the FESEM images showed that DNHK loaded PLGA microparticles containing 4% of MTX was considered the best system because of the absence of aggregates (Figure 2(B)).

There are many factors that can influence the performance of the obtainment of nanoparticles, thus changing the specific characteristics, such as the size and shape. The choice of the method for drying the nanoparticles and the used equipment parameters are strongly related to the morphology of the nanoparticles. The selection of the correct temperature is important for obtaining spherical nanoparticles, which is direct linked to drug controlled release potential of these systems (Oliveira et al. 2013).

Drug delivery vehicles introduced in the blood stream undergo a complex journey prior to arriving at the target site (Langer 1990; Yoo et al. 2011). Various physicochemical attributes of drug carriers, such as composition, size, surface chemistry, shape and mechanical flexibility play important roles in their therapeutic function (Doshi and Mitragotri 2009; Mitragotri and Lahann 2009; Yoo et al. 2011). Particles in the size range of nanometers to a few microns have been used for intravenous applications (Ilium et al. 1982; Muro et al. 2008). The upper limit of the particle size that can be used for intravenous applications is limited by the smallest capillaries which are only a few microns in diameter (Yoo et al. 2011). In addition, the particle size also impacts on the extension of cellular uptake by phagocytosis and endocytosis. Particles in nanoscale are usually internalized by endocytosis, whereas particles in microscale are believed to be internalized by phagocytosis (Rejman et al. 2004). The obtained particles diameter was 215 nm, and it is suggested that it can be administered by intravenous route, as well as it may be internalized by endocytosis.

The particles are nanometric and can be administered by parenteral route. In addition, a delayed schistosomicidal activity of incorporated (−)-6,6′-dinitrohinokinin was due to the controlled release from PLGA nanoparticles. Advantages of the incorporation will be better evaluated by *in vivo* studies.

### X-ray diffraction

**Figure 3.**
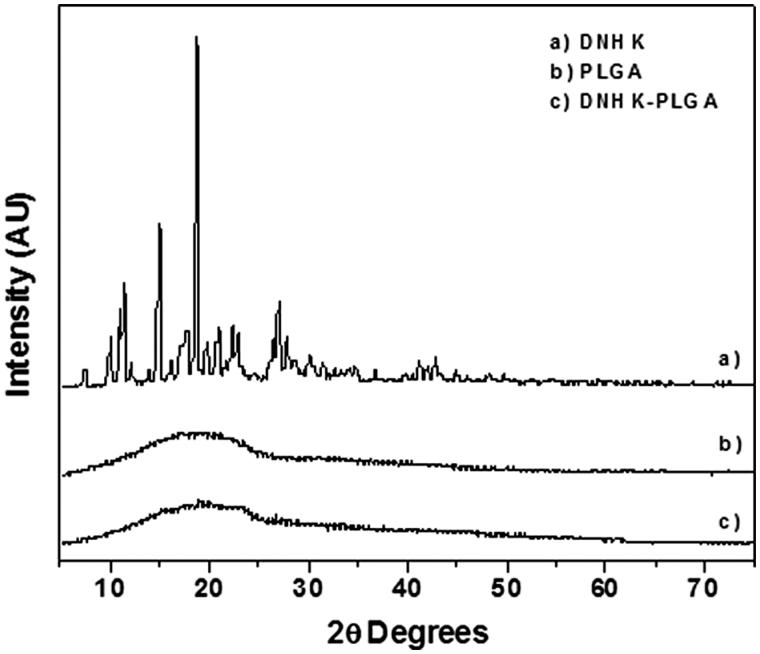
X-ray pattern obtained for (a) DNHK, (b) PLGA and (c) DNHK-loaded PLGA nanoparticles.

Regarding the XRD patterns, a broad peak centred at ca. 19° was observed for unloaded and loaded PLGA nanoparticles assigned to the presence of amorphous phase. The absence of the typical peaks of DNHK for the loaded PLGA indicates that DNHK was well dispersed in the polymeric matrix, and that it did not crystallize because of the presence of the amorphous polymer (Figure 3) (Bae et al. 2009).

### *In vitro* schistosomicidal activity of DNHK

In the last year, several research groups have been studying new alternatives in the treatment of schistosomiasis (Allam et al. 2013). However, few studies have been performed with drugs loaded in polymeric nanoparticles against *Schistosoma* species. Luz et al. (2012) showed the effect of curcumin-loaded PLGA nanoparticles. In another study, De Araújo et al. (2007) reported the *in vitro* schistosomicidal activity of the nanoemulsion containing 2-(butylamino)-1-phenyl-1-ethanethiosulfuric acid (BphEA).

In this study, we evaluated the effects of incubation with different concentrations of DNHK-loaded PLGA on the motor activity, pairing and eggs production of adult worm pairs. The adult worms incubated with praziquantel at 10 µM (positive control) at 24 and 120 h showed an absence of movement, which indicated the death of parasites. *S. mansoni* adult worms incubated with DNHK-loaded PLGA at concentrations higher than 150 µM at 24 h displayed a similar effect in comparison with the positive control group (Table 1). At concentration of 100 µM the adult worms showed no motility (dead) in 120 h. The negative control with parasites in RPMI plus PLGA did not show alteration on motor activity. DNHK-loaded PLGA afforded LC_50_ values of 137.0 ± 2.12 µM and 79.01 ± 1.90 µM at 24 and 120 h, respectively (Table 1) against female and male *S. mansoni* worms, respectively. Pereira et al. (2015) reported the effect of DNHK-free on adult worms of *S. mansoni*, and the LC_50_ value was 103.9 ± 3.6 µM at 24 h.

**Table 1.** Effect of DNHK-loaded PLGA nanoparticles against *S. mansoni* adult worms pairs.

Groups	Incubation time (h)	Unpaired worms (%)	Dead worms (%)
RPMI 1640	24	0	0
	120	0	0
RPMI 1640 + PLGA	24	0	0
	120	0	0
PZQ 10 µM	24	0	100 ± 0***
	120	0	100 ± 0***
10 μM	24	0 ± 0	0 ± 0
	120	0 ± 0	0 ± 0
50 μM	24	0 ± 0	0 ± 0
	120	100 ± 0***	0 ± 0
100 μM	24	0 ± 0	0 ± 0
	120	100 ± 0***	100 ± 0***
150 μM	24	100 ± 0***	100 ± 0***
	120	100 ± 0***	100 ± 0***
200 μM	24	100 ± 0***	100 ± 0***
	120	100 ± 0***	100 ± 0***

Asterisk indicates statistically significant differences compared with the negative control group (***p < 0.001). All experiments were carried out in quadruplicate and repeated at least three times.

Other parameters evaluated in this study were the separation of adult worm couples and egg production (Table 1). Besides playing a role in the pathological aspects of schistosomiasis, the eggs are crucial for the transmission of this infection (Freitas et al. 2007). The DNHK-loaded PLGA nanoparticles caused a separation of 100% of *S. mansoni* couples at concentrations of 50 and 100 μM in 120 h, and at concentrations of 150 and 200 µM in 24 h. Also, it was observed a reduction of the total eggs number in 120 h at the same concentrations (data not shown). In the negative control groups, it was not observed separation and alteration in number of eggs (Table 1).

## Conclusions

The purity of DNHK was estimated to be 95%, and its encapsulation process into PLGA particles was successfully achieved by the nanoprecipitation method. The FESEM technique revealed the production of particles spherically shaped with an average diameter of 215 nm and a PI of 0.102, as determined by laser light scattering. From the obtained results it can be assured that DNHK was incorporated in monodisperse nanospheres of PLGA. The encapsulation efficiency of DNHK in PLGA nanoparticles was 42.2%, which can be considered satisfactory taking into account the particle size. The produced nanometric diameter of the DNHK-loaded PLGA 85:15 nanoparticles allows its intravenous route administration in the case of a possible schistosomiasis treatment. Biological studies are underway for assessment of the schistosomicidal activity of this novel system *in vivo*, as well as further DNHK release studies from the obtained nanoparticles. In addition, a delayed schistosomicidal activity of incorporated (−)-6,6′-dinitrohinokinin was due to the controlled release from PLGA nanoparticles.

## Supplementary Material

Marcio_Silva_et_al_et_al_supplemental_content.zip
