# Supplementary material for: In vitro schistosomicidal activity of the lignan (−)-6,6′-dinitrohinokinin (DNHK) loaded into poly(lactic-co-glycolic acid) nanoparticles against Schistosoma mansoni
Source: Pharm Biol. 2017 Nov 26;55(1):2270–6. doi: 10.1080/13880209.2017.1405996 (PMC6130564; doi:10.1080/13880209.2017.1405996)
Supplement: Marcio_Silva_et_al_et_al_supplemental_content.zip [file IPHB_A_1405996_SM7794.zip › Marcio Silva et al et al supplemental content.doc]

**Supplementary Information**

*In vitro* schistossomicidal activity of the lignan (-)-6,6’-dinitrohinokinin (DNHK) loaded into poly(lactic-co-glycolic acid) nanoparticles against *Schistosoma mansoni*

Thaís C. Lima^a^, Rodrigo Lucarini^a^, Priscilla P. Luz^b^, Emerson H. de Faria^a^, Liziane Marçal^a^, Lizandra G. Magalhães^a^, Fernanda R. Badoco^a^, Viviane R. Esperandim^a^, Eduardo F. Molina^a^, Rosangela S. Laurentz^c^, Regiane G. Lima^c^, Wilson R. Cunha^a^, Jairo K. Bastos^d^, Marcio L. A. e Silva^a(*)^

^a^Núcleo de Ciências Exatas e Tecnológicas, Universidade de Franca - Av. Dr. Armando Salles Oliveira, 201, 14404-600, Franca-SP, Brazil; ^b^Universidade Federal do Espírito Santo (UFES), Departamento de Química – CCE, Av. Fernando Ferrari, 514 – Goiabeiras, Vitória, ES - CEP: 29060-900; ^c^Faculdade de Engenharia de Ilha Solteira, Universidade Estadual Paulista- Avenida Brasil, 56, 15385-000, Ilha Solteira-SP, Brazil; ^d^School of Pharmaceutical Sciences of Ribeirão Preto, University of São Paulo - Avenida do Café S/N, 14040-903, Ribeirão Preto-SP, Brazil;

Figure S1. HPLC chromatogram of the synthesized DNHK.

**Figure S2**. ^1^ H NMR (400 MHz, DMSO-*d_6_*) spectrum of DNHK in CDCl_3_

**Figure S3**. ^13^C NMR (100 MHz, DMSO-*d_6_*) spectrum of DNHK in CDCl_3_

**Table S2.** Shows the DNHK molecule and the characterization data

|  | Yield 97.6 %; –29.07° (*c* 0,008, CHCl_3_, *ee* > 99%); mp 191-193 °C; ^1^H NMR (300 MHz, CDCl_3_) *δ* 7.5 (s, 1H); 7.48 (s, 1H); 6.8 (s, 1H); 6.6 (s, 1H); 6.1 (m, 2H); 4.35 (dd, 1H, J = 7.1 Hz and J = 9.1 Hz); 4.0 (dd, 1H, J = 7.3 Hz and J = 9.1 Hz); 3.26 (d, 2H, J = 7.1 Hz); 3.2 (dd, 1H, J = 6.3 Hz and J = 13.6 Hz); 3.0 (dd, 1H, J = 7.8 Hz and J = 13.6 Hz); 2,8 (m, 2H); ^13^C NMR (75 MHz, CDCl_3_): 178.00, 152.3, 152.2, 147.6, 143.1, 142.9, 130.9, 130.7, 112.5, 111.2, 106.6, 106.2, 103.6, 103.5, 71.4, 45.7, 41.7, 37.2, 34.2  [  ]  25  *D*  *α* |
| --- | --- |
